# Epimorphin-Induced MET Sensitizes Ovarian Cancer Cells to Platinum

**DOI:** 10.1371/journal.pone.0072637

**Published:** 2013-09-09

**Authors:** Kok-Hooi Yew, Jennifer Crow, Jeff Hirst, Ziyan Pressetto, Andrew K. Godwin

**Affiliations:** 1 Department of Pathology and Laboratory Medicine, University of Kansas Medical Center, Kansas City, Kansas, United States of America; 2 University of Kansas Cancer Center, Kansas City, Kansas, United States of America; University of Kentucky, United States of America

## Abstract

Distinctive genotypic and phenotypic features of ovarian cancer via epithelial-mesenchymal transition (EMT) have been correlated with drug resistance and disease recurrence. We investigated whether therapeutic reversal of EMT could re-sensitize ovarian cancer cells (OCCs) to existing chemotherapy. We report that epimorphin, a morphogenic protein, has pivotal control over mesenchymal versus epithelial cell lineage decision of the putative OCCs. Exposure to epimorphin induced morphological changes reminiscent of mesenchymal-to-epithelial transition (MET), but in a dose dependent manner, i.e., at 10 µg/mL of epimorphin cells obtain a more mesenchymal-like morphology while at 20 µg/mL of epimorphin cells display an epithelial morphology. The latter changes were accompanied by suppression of mesenchymal markers, such as vimentin (∼8-fold↓, *p*<0.02), Twist1 (∼7-fold↓, *p*<0.03), dystroglycan (∼4-fold↓, *p*<0.01) and palladin (∼3-fold↓, *p*<0.01). Conversely, significant elevations of KLF4 (∼28-fold↑, *p*<0.002), β-catenin (∼6-fold↑, *p*<0.004), EpCAM (∼6-fold↑, *p*<0.0002) and occludin (∼15-fold↑, *p*<0.004) mRNAs as part of the commitment to the epithelial cell lineage were detected in response to 20 µg/mL of exogenous epimorphin. Changes in occludin mRNA levels were accompanied by a parallel, albeit weaker expression at the protein level (∼5-fold↑, *p*<0.001). Likewise, acquisition of epithelial-like properties, including mucin1, CK19, and β-catenin gene expression, was also obtained following epimorphin treatment. Further, MMP3 production was found to be reduced whereas laminin secretion was strongly amplified upon epimorphin-induced MET. These results suggest there is a dosage window for actions of epimorphin on cellular differentiation, wherein it can either suppress or enhance epithelial differentiation of OCCs. Importantly, induction of epithelial-like phenotypes by epimorphin led to an enhanced sensitivity to carboplatin. Overall, we demonstrate that epimorphin can revert OCCs away from their mesenchymal phenotype and toward an epithelial phenotype, thereby enhancing their sensitivity to a front-line chemotherapeutic agent.

## Introduction

Epithelial ovarian cancer (EOC) is a leading cause of death in women, often due to late-stage recognition and aggressive tumor relapse. High patient morbidity is attributable in part to recurrent growth of residual ovarian tumor cells that become resistant to standard chemotherapeutic treatments, and then aggressively proliferate and spread or metastasize to multiple sites. Recent studies have revealed that the ovarian cancer cells with epithelial features are in fact more sensitive to chemotherapy regimens as compared to tumor cells with mesenchymal traits [1], [2], [36]. However, when these cells undergo epithelial-to-mesenchymal transition (EMT), they become less responsive to conventional therapeutic regimens [1]–[3], [36]. EMT, induced by a wide array of stimuli and cytokines, has become prominently implicated as a means by which differentiated EOCs undergo a phenotypic conversion that is associated with the loss of epithelial characteristics (e.g., occludin) and the acquisition of mesenchymal markers (e.g., vimentin). Therefore, reversal of EMT (i.e., MET) may render tumor cells more susceptible to first-line anti-cancer drugs and potentiate the apoptotic effects of certain chemotherapeutic agents.

Epimorphin, also known as syntaxin-2, is an important mediator of numerous fundamental processes in embryonic development, and frequently play a crucial role in the region of mesenchyme-epithelium interactions [4]–[8]. Subsequent studies have also shown that epimorphin mediates epithelial patterning and morphogenesis in many tissues, such as pancreatic ducts [5], mammary gland [6], lung [7], and small intestinal epithelium [8]. While epimorphin appears to be expressed in the mammalian ovary and testis [9], the precise mechanism by which this mesenchymal morphogen exerts its regulatory effect on human gonads remains largely unknown.

Although the cognate epimorphin receptor and its downstream signaling mechanism have not yet been identified, epimorphin has been found to bind to αV integrin receptors on mammary epithelial cells [10] and EGF receptors on intestinal epithelial cells [11] which activate transcription factors CCAAT/enhancer binding protein (C/EBPα and β) [12], [15] and NF-κB [13]. Interestingly, krueppel-like factor 4 (KLF4), a zinc-finger transcription factor expressed in epithelial cells of several organs [16], has been shown to bind directly to the C/EBPβ promoter [17] and interact with NF-κB subunit p65 [18], which regulate lung epithelial differentiation during organogenesis [19] and mammary epithelial branching [20], respectively. Further investigations have indicated that epimorphin can induce epithelial morphogenesis by regulating urokinase type plasminogen activator (uPA) [13], [14] as well as involucrin and cytokeratin [15].

Given the proposed role of epimorphin as a pro-epithelial factor in various tissues [5]–[8], we evaluated whether epimorphin signaling may be able to induce the ovarian cancer cells with mesenchymal traits toward the epithelial cell lineage and whether this conversion could lead to enhanced sensitivity to standard chemotherapy agents.

## Materials and Methods

### Reagents

Fetal Bovine Serum, SYBR Green, Reference Dye for Quantitative PCR, and Protease Inhibitor Cocktail were obtained from Sigma-Aldrich (St. Louis, MO). RPMI Medium 1640 was obtained from Gibco/Invitrogen. RNeasy Mini Kit, Sensiscript® Reverse Transcriptase Kit, QIAamp DNA Micro Kit, and QIAquick Gel Extraction Kit were all purchased from Qiagen (Valencia, CA). AmpliTaq Gold with GeneAmp 10X PCR Buffer and MgCl_2_ solution were obtained from Applied Biosystems (Foster City, CA).

### Cell Culture and Treatment

The human ovarian cancer cells (OCCs), A1847, A2780 and OVCAR10 have been described previously [21] and were used in all experiments. Cells were grown in RPMI-1640 media supplemented with 10% (v/v) fetal calf serum, 2 mm l-glutamine, 0.2 units/ml human insulin, 50 units/ml penicillin, 50 μg/ml streptomycin at 37°C under a humidified condition of 95% air and 5% CO_2_. A1847 and A2780 were plated at a density of 5×10^4^ cells/mL in 24-well plates and were treated with either 10 µg/mL or 20 µg/mL of epimorphin (R&D Systems, Minneapolis, MN) followed by 3 days of incubation in 1% serum-containing medium.

### SYBR Green Real-Time Quantitative PCR

Total RNA was extracted from epimorphin-treated and untreated OCCs and digested with DNase. RNA was subjected to reverse transcription. cDNA was then amplified using a Bio-Rad CFX96™ (Hercules, CA) real-time detection system. Unless otherwise specified, each reaction mixture contained 10X Gold Buffer, 25 mm MgCl_2_, 2.5 mM dNTPs, 10X SYBR Green, AmpliTaq Gold polymerase, Reference Dye, dH_2_O, DNA template and 10 µM of each primer. Amplification was performed by initial polymerase activation for 10 min at 95°C, and 40 cycles of denaturation at 95°C for 15 sec, annealing at 60°C for 20 sec and elongation for 30 sec at 72°C (Table S1). The fluorescence threshold value was calculated using the CFX96™ real-time system software. The calculation of relative change in mRNA levels was performed using the delta-delta method [22], with normalization for the housekeeping gene RPL13A.

### Fluorescence Activated Cell Sorting (FACS)

Epimorphin-treated and untreated ovarian cancer cells were fixed with 2% phosphate-buffered paraformaldehyde for 30 min at RT. After fixation, the cells were permeabilized with 1X FACS™ permeabilizing Solution 2 (BD Biosciences, San Jose, CA) for 30 min at RT, blocked with bovine serum albumin for 30 min and then incubated with FITC-conjugate mouse anti-human-EpCAM (BD Biosciences, San Jose, CA) and FITC-conjugate mouse anti-human-vimentin (Abcam, Cambridge, MA) in the dark for 30 min at RT. The cells were rinsed with PBS and fluorescence digital images were captured using BD Biosciences LSR II. BD FACSDiva™ Version 6 software was applied to characterize and monitor performance of the cytometers. FlowJo software was then utilized for the analysis of flow cytometry data. Single-stained controls were used to set compensation parameters and unstained samples used to set negative gates.

### Immunostaining

Untreated and epimoprhin-treated cells cultured on non-coated glass coverslips (Fisher Scientific, Pittsburgh, PA) at a density of 4×10^4^ cells were fixed with ethanol for 30 min at 4°C. After fixation, the cells were permeabilized with cold (−20°C) acetone for 3 min at room temperature, blocked with 1% bovine serum albumin for 1 hour, and then incubated with β-catenin (L54E2) mouse mAB (Alexa Fluor® 488 conjugate; Cell Signaling Tech., Danvers, MA) in a moist chamber overnight at 4°C. On the next day, the cells were rinsed with PBS. After several washes, coverslips containing cells were mounted onto slides in VECTASHIELD® mounting medium with DAPI (Vector Labs, Inc, Burlingame, CA). Fluorescence digital images were captured using a Nikon Eclipse 80*i* (Melville, NY) microscope attached with a Nikon Q-imaging camera adaptor. MetaMorph Image Analysis software (version 7.7.0.0) was used to acquire and analyze images.

### SDS-PAGE and Western Blot Analysis

Proteins were separated on a 10% Tris-HCl Ready Gel (Bio-Rad, Hercules, CA), transferred onto nitrocellulose membranes, and incubated with mouse monoclonal [ab6276] to β-actin at a dilution of 1/5000, mouse monoclonal [ab28081] to mucin-1 at a dilution of 1/1000, mouse monoclonal [ab7754] to cytokeratin 19 (CK-19) at a dilution of 1/1000 or rabbit polyclonal [ab31721] to occludin at a dilution of 1/250 for 2 hr at RT. All western blot antibodies were obtained from ABCAM (Cambridge, MA). After incubation, the membranes were washed 3X for 15 min in washing buffer (PBS-0.05% Tween 20) and incubated with a secondary anti-mouse (β-actin, mucin-1, vimentin, palladin, and cytokeratin 19) or anti-rabbit (occludin) antibody coupled to horseradish peroxidase (Vector Labs, Burlingame, CA) for 1 hr at RT. Then, the membranes were washed 3X for 15 min in washing buffer, and immunoreactivity was normalized by chemiluminescence (Amersham, ECL+Plus Kit) according to the manufacturer's instructions. The membranes were exposed to Kodak scientific imaging films (Rochester, NY) within 1 min for detection. Pixel densities of blot images were calculated using Image-J software (NIH). Changes in protein levels were normalized to controls and expressed as fold change relative to controls.

### Measurement of Cytokines

To screen for epimorphin-induced extracellular matrix secretion, A1847 OCCs (4×10^4^ cells/ml in 24-well plates) were incubated with media alone (negative control), 10 μg/mL or 20 μg/mL of epimorphin for 3 days. Laminin (Millipore, Temecula, CA) and MMP3 (R&D Systems, Minneapolis, MN) secretion into the culture supernatants were measured by sandwich ELISA according to the protocol from the manufacturers. Data points are expressed as the mean concentration of duplicate assays at 450 nm.

### Drug Treatments, Cell Viability Assay and Nexin Cell Death Assay

A1847, A2780 & OVCAR10 were plated at a density of 2×10^4^ cells/mL in 48-well plates and were cultured with epimorphin at a concentration of 20 µg/mL for 3 days. Epimorphin-treated and untreated cells were then cultured with a serial dose of carboplatin (Selleck Chemicals, Houston, TX, USA) for additional 3 days. Carboplatin dose range for epimorphin-treated and untreated A1847 were 1 µM, 10 µM, 50 µM, 100 µM, 200 µM, and 400 µM. Carboplatin dose range for epimorphin-treated and untreated A2780 were 1 µM, 10 µM, 50 µM, 100 µM, 150 µM, and 200 µM. Carboplatin dose range for epimorphin-treated and untreated OVCAR10 were 50 µM, 100 µM, 150 µM, 200 µM, 400 µM, and 500 µM. Cell viability was determined by measuring metabolic activity using CellTiter-Blue® by Promega and plates were imaged on the Tecan Fluorometer. Data were then normalized to percentage inhibition and IC_50_ concentrations of carboplatin were determined for epimorphin-treated and untreated cells by the SigmaPlot graphing program (Systat Software). Cell death or apoptosis was quantitated using the Guava Nexin Annexin V assay via a Guava EasyCyte HT flow cytometer (Guava Technologies, Hayward, CA). The Nexin assay uses two dyes: 7-AAD, a cell impermeant dye, as an indicator of membrane structural integrity and Annexin V-PE to detect phosphatidylserine on the external membrane of apoptotic cells. Samples were prepared as per the manufacturer's specifications. Data were normalized to the controls and are represented as means ± S.D.

### Statistical Analysis

Data were analyzed using the Microsoft Office Excel 2010 and expressed as means ± S.D. where appropriate. Two group comparisons were evaluated using the unpaired Student's t-test. Values of p<0.05 were considered statistically significant.

## Results

### Morphological and Molecular Effects of Epimorphin Associated with MET in Ovarian Cancer Cells

**Figure 1:**
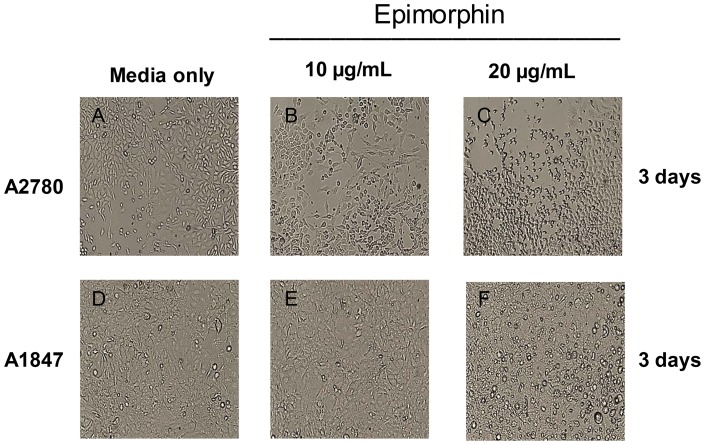
Exogenous epimorphin induces a dose-dependent change in ovarian cancer cell morphology. A2780 and A1847 were incubated with 10 µg/mL or 20 µg/mL of epimorphin for 3 days. A–F: Epimorphin (20 µg/ml) induced a rounded cobblestone, epithelial-like appearance in both A2780 (C) and A1847 (F). In comparison, epimorphin at 10 µg/ml lead to a more elongated mesenchymal morphology in both A2780 (B) and A1847 (E) compared with untreated controls (A&D). Images of cell morphology were captured at 10X magnification, with at least 3 images per well, using an upright phase-contrast microscope (T3.15A; Fisher Scientific).

**Figure 2:**
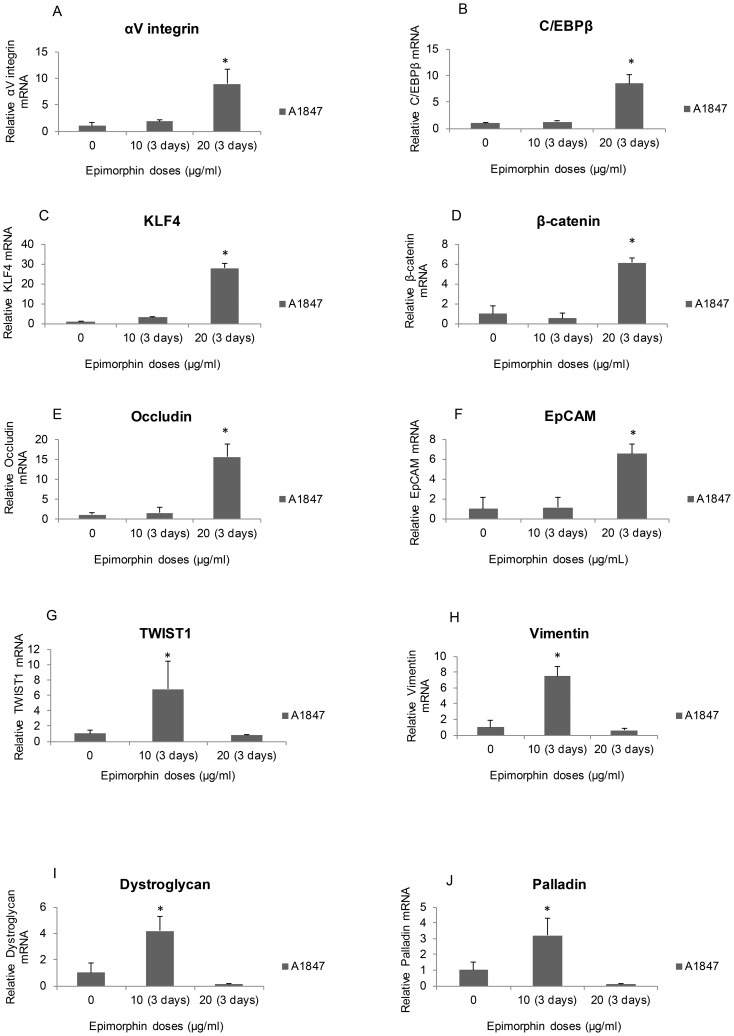
Quantitative effects of epimorphin on expression of epithelial and mesenchymal genes in ovarian cancer cells. A and B: αV-integrin receptor (A) and C/EBPβ (B) showed marked elevation of mRNA levels in response to 20 µg/mL of epimorphin. C–F: Epithelial markers such as KLF4 (C), β-catenin (D), occludin (E), and EpCAM (F) were found to be upregulated by exogenous 20 µg/mL of epimorphin. G–J: Expression of mesenchymal markers TWIST1 (G), vimentin (H), dystroglycan (I) and palladin (J) were downregulated following treatment with epimorphin (20 µg/ml). However, all four mesenchymal markers (G–J) were found to be upregulated by 10 µg/mL of epimorphin (means ± S.D., n = 3) [*p<0.05 compared with control].

Previous works have implicated epimorphin as mediators of epithelial morphogenesis in various organs and cells [4]–[13]. Nevertheless, little is known about the mechanism by which epimorphin mediate such effects in ovarian cancer cells. We first utilized an ELISA Kit to compare the levels of epimorphin in various ovarian cancer cell lines with adipose-derived mesenchymal stem cells which were used as positive controls. The endogenous epimorphin production was relatively low (range: 0.1–0.3 ng/ml) across all ovarian cancer cell lines tested compared to adipose-derived mesenchymal stem cells (range: 10–12 ng/ml) (data not shown). We then determined the optimum time and dose of epimorphin treatment to induce MET in the ovarian cancer cell line, A2780. Recent studies have shown that 20 ug/ml of epimorphin was able to activate MEK-ERK1/2 pathway (11) and induce MMPs differentiation (13). To assess the dose dependent response in ovarian cancer cells, we first performed a broad dose-response (2 to 20 ug/ml) of epimorphin using A2780 cells. We found after 3-day at 20 µg/mL of epimorphin, A2780 cells underwent significant morphological changes (from mesenchymal to epithelial) as analysed by phase contrast light microscopy, immunoblotting and real-time quantitative RT-PCR whereas 2 ug/ml of epimorphin elicited no cellular responses (data not shown). We next evaluated the optimal dose of epimorphin and found that both A2780 and A1847 treated with the lower dose (10 µg/mL) of epimorphin exhibited a more stellate and elongated mesenchymal-like appearance (Fig. 1B & 1E). In comparison, the higher dose (20 µg/mL) lead to a more rounded epithelial-like shaped as visualized by phase contrast microscopic examination (Fig. 1C & 1F). These results suggested a biphasic effect of epimorphin and a narrow dosage window to optimally revert mesenchymal cells to a more epithelial morphology. Furthermore, we removed the exogenous epimorphin by replacing the cell culture with fresh media upon epimorphin-induced MET for an additional 3-day incubation. The epimorphin-induced MET of ovarian cancer cells remained epithelial phenotype as analyzed by a phase contrast light microscopy and real-time quantitative PCR (data not shown). To further analyze the nature of this effect, we measured the levels of αV-integrin, an “epimorphin receptor” in A1847 and found that the steady-state levels of αV-integrin were low but were induced 9-fold by 20 µg/mL epimorphin (Fig. 2A). Similar to αV-integrin receptor levels, the downstream mediators, e.g., transcription factors C/EBPβ (∼9-fold↑, *p*<0.056) (Fig. 2B) and KLF4 (∼28-fold↑, *p*<0.002) (Fig. 2C), were found to be significantly elevated, suggesting that C/EBPβ and KLF4 may be key regulators of epimorphin mediated MET. Similarly, mRNA levels of β-catenin (∼6-fold↑, *p*<0.004) (Fig. 2D), occludin (∼15-fold↑, *p*<0.004) (Fig. 2E) and EpCAM (∼6-fold↑, *p*<0.0002) (Fig. 2F) were found to be strongly upregulated at the highest dose of epimorphin, again indicating supporting conversion to an epithelial-like phenotype. Molecular markers of mesenchymal phenotype, such as TWIST1 (Fig. 2G), vimentin (Fig. 2H), dystroglycan (Fig. 2I) and palladin (Fig. 2J), were suppressed by 20 µg/mL epimorphin but increased significantly upon stimulation of A1847 with a lower dose of epimorphin, implying that the switch between mesenchymal and epithelial phenotypes is likely to be dose-dependent.

### Effects of Epimorphin on Gene Expression Phenotypes in A1847

**Figure 3:**
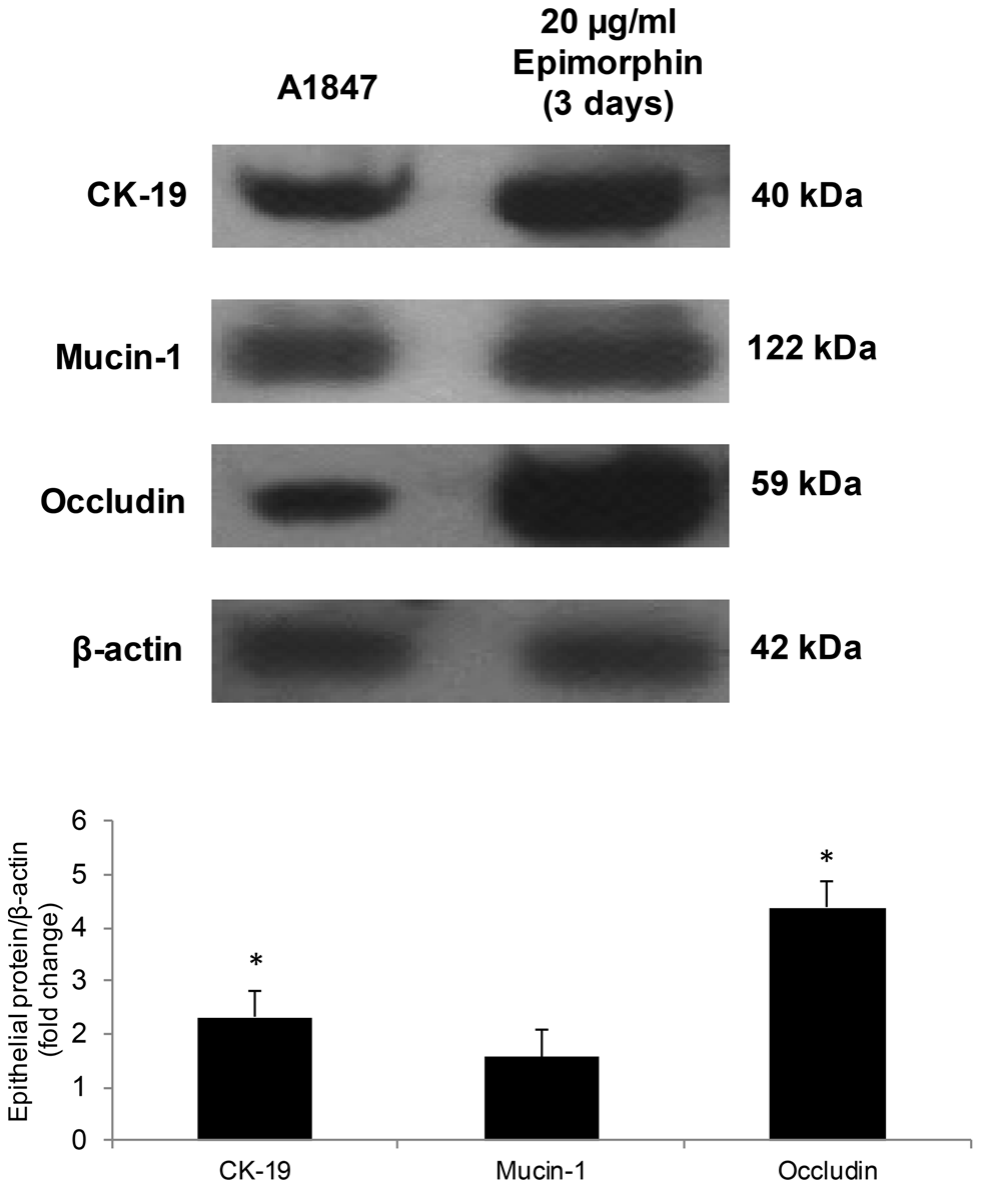
Analysis of expression of MET-associated proteins. Protein expression of the epithelial-associated markers, e.g., mucin-1, CK-19 and occludin were evaluated by immunoblotting and band densities were quantified by Image-J software. Fold increases in protein expression of treated (20 µg/ml epimorphin) versus untreated A1847: 2.5-fold increase for CK-19; 1.5-fold increase for mucin-1, and 4.5-fold increase for occludin. β-actin served as the loading control. (means ± S.D., n = 3). [*p<0.05 compared with control].

**Figure 4:**
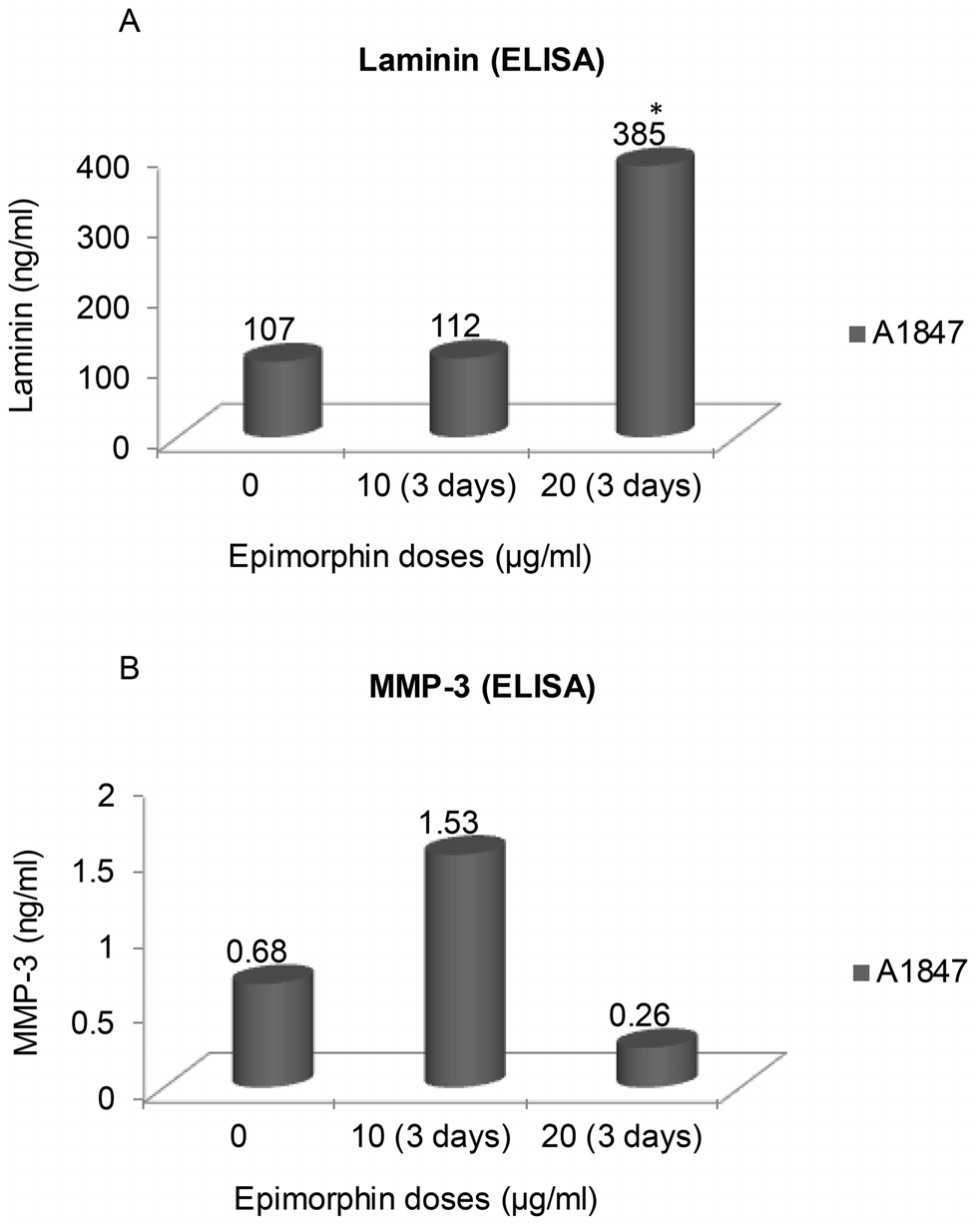
Evaluation of laminin and MMP-3 following epimorphin induced MET. Secretion of laminin (epithelial marker) and MMP-3 (mesenchymal marker) was measure by ELISA following treatment of A1847 ovarian cancer cells with epimorphin for 3 days (at 10 or 20 µg/ml). Exposure to epimorphin (20 µg/ml) significantly increased laminin production and lead to a decrease in MMP-3 secretion when compared to control. In comparison, MMP-3 release was enhanced when A1847 treated with the lower concentration of epimorphin (10 µg/ml), again suggesting epimorphin can induce phenotypic changes in ovarian cancer cells in a dose-dependent manner (means ± S.D., n = 3) [*p<0.05 compared with control].

In an effort to further confirm the inductive effects of epimorphin on phenotypic modifications, immunoblotting and ELISA were performed to determine the protein expression profile of both epithelial and mesenchymal markers. Quantitative analysis of occludin protein in A1847 showed that treatment with higher-dose epimorphin increased occludin expression by five-folds (Fig. 3) compared to untreated, consistent with the result seen with the mRNA levels (Fig. 2E). Likewise, similar increases were obtained for CK-19 and mucin-1 (Fig. 3), again implying that epimorphin-treated A1847 undergo phenotypic changes associated with MET. However, the expression of mesenchymal genes, palladin and vimentin (Figure S1) increased by 1- and 2-fold, respectively, in A1847 treated with 10 µg/ml epimorphin, again suggesting that the induction of EMT or MET in response to epimorphin is bidirectional depending on the applied dose. We next performed ELISA assays to determine release of proteins associated with epithelial and mesenchymal phenotypes. Interestingly, secretion of laminin (Fig. 4A), an extracellular glycoprotein essential for the development of epithelial cell polarity, was found to be elevated significantly upon treatment of A1847 with epimorphin. Conversely, production of MMP3 (Fig. 4B), a mesenchymal marker important for cancer cell invasion and migration, was found to be decreased after 20 µg/mL epimorphin treatment but strongly upregulated at 10 µg/mL. Taken together, these data demonstrate that there is progressive commitment of ovarian tumor cells toward an epithelial lineage when cultured with a sufficient dose of epimorphin.

### Epithelial Differentiation in Response to Exogenous Epimorphin

**Figure 5:**
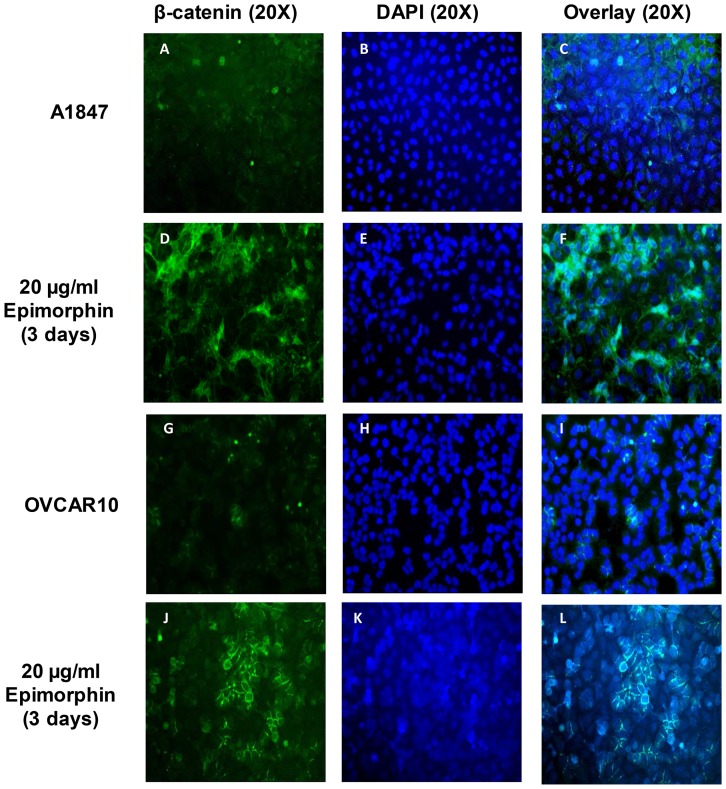
β-catenin activation of A1847 in response to epimorphin. Untreated and 20 µg/mL epimorphin-treated A1847 and OVCAR10 were analyzed for β-catenin, a marker of epithelial differentiation, by immunostaining. β-catenin (green), DAPI (blue) and merge (neon green) in untreated A1847 (A–C) and OVCAR10 (G–I); epimorphin-treated A1847 (D–F) and OVCAR10 (J–L). Immunostaining analysis showed increased expression of β-catenin-positive cells in epimorphin-treated A1847 (D&F) and epimorphin-treated OVCAR10 (J&L). There were abundant β-catenin positive cells at and around the cell-cell junctions of epimorphin-induced A1847 and OVCAR10 (F&L) compared to untreated controls (C&I).

β-catenin, a key factor in the Wnt signaling pathway, has been shown to determine epithelial cell fate during embryonic development of the ovary [23]. Loss of β-catenin late in embryonic development resulted in profound defects in epithelial maturation at adulthood [24]. To further investigate whether the epimorphin-mediated MET is associated with increased endogenous expression of β-catenin, we utilized immunostaining for β-catenin to assess the epithelial cell differentiation of epimorphin-treated A1847. Both untreated A1847 and OVCAR10 had few β-catenin-positive cells; but showed prominent expansion of the region at and around the cell-cell junctions, most notably in epimorphin-treated A1847 and OVCAR10 (Fig. 5). An increase in β-catenin-positive cells seen in epimorphin-treated A1847 and OVCAR10 represented a form of dose-response of these cells to 20 µg/mL epimorphin. This experimental evidence points to a role for epimorphin signaling in the induction of β-catenin differentiation which may be an important determinant of epithelial lineage commitment in ovarian tumor cells. Furthermore, FACS analysis identified a sub-fraction of A1847 cells with upregulated EpCAM (epithelial marker) and downregulated vimentin (mesenchymal marker) following treatment with 20 µg/mL epimorphin (Figure S2 & S3).

### Cell Viability and Annexin-V Activity during Carboplatin-induced Apoptosis in Epimorphin-treated OCCs

**Figure 6:**
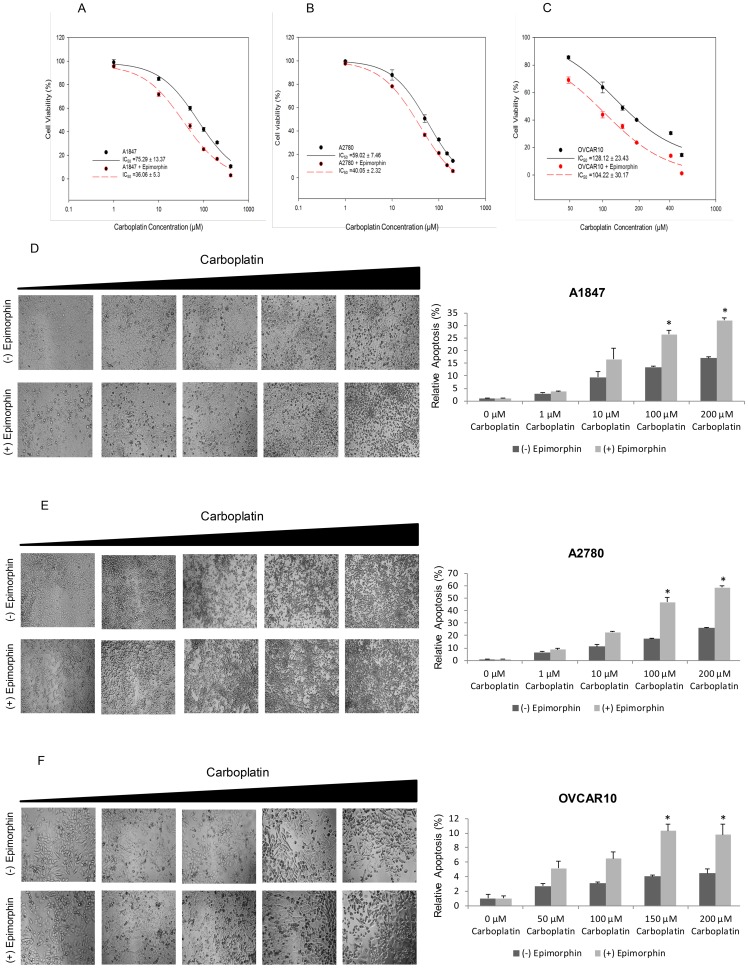
Carboplatin-induced changes in cell viability and apoptosis following epimorphin-induced MET. A-F: A1847, A2780, and OVCAR10 were treated with 20 µg/mL epimorphin for 3 days. After 3 days, epimorphin-treated and untreated OCCs were cultured in triplicate with serial doses of carboplatin for an additional 3 days. Cell viability was quantified using a CellTiter Blue® assay (A–C). Apoptosis was quantitated using a Guava Nexin assay (D–F). A–C: IC_50_ values indicate carboplatin induced more cell viability loss in all three epimophin-treated OCCs than the untreated controls in a dose-dependent manner. D–F: Detection of apoptotic responses was found to increase in all three epimorphin-treated OCCs with increasing concentrations of cisplatin compared to those of the untreated controls. Data were normalized to the controls and are represented as means ± S.D. [*p<0.05 compared with control]. Images of apoptotic cells were captured at 10X magnification, with at least 3 images per well, using an upright phase-contrast microscope (T3.15A; Fisher Scientific).

To determine whether carboplatin can preferentially kill ovarian cancer cells with more epithelial-like morphologies, we compared the ability of carboplatin to induce cell death following epimorphin-treatment. As shown in Figure 6, carboplatin exposure lead to a significant decrease in the number of viable cells following epimorphin induced MET for A1847 (*p* = 0.005) (Fig. 6A) and A2780 (*p* = 0.007) (Fig. 6B) as compared to controls. Although the trend was similar, the change in sensitivity to OVCAR10 (Fig. 6C) with or without epimorphin was not statistically different (*p* = 0.170), possibly due to the lack of dose scale at the higher end of the range for plotting and modeling the response data. Fluorochrome-labeled Annexin V was used to identify apoptotic cells [25], [26]. As shown in Figure 6D–6F, induction of apoptosis was observed in the three epimorphin-treated OCCs, including the more carboplatin-resistant cell line, OVCAR10 as compared with the untreated OCCs. The percentage of apoptotic cells increased in epimorphin-treated cells with increasing concentrations of carboplatin (Fig. 6D–6F). These results suggest that the OCCs with mesenchymal features become more sensitive to carboplatin-induced apoptosis after epimorphin associated differentiated to an epithelial-like state. Importantly, when evaluated using 10 µg/ml of epimorphin, which fails to induced MET, the three ovarian cancer cell lines evaluated (i.e., A1847, A2780 and OVCAR10) showed increased resistance to carboplatin as measured by CellTiter® Blue (Figure S4). These results emphasize that reversal of EMT may serve to help further sensitize recurrent disease to frontline therapies. Since elevated cell proliferation could dramatically impact carboplatin resistance and platinum drugs only damage cells during S-phase of the cell cycle, manual cell counts using a hemocytometer have been performed to monitor proliferation at the end of the culture. Little-to-no change on cell growth rates was observed for epimorphin-treated as compared to untreated OCCs (data not shown).

## Discussion

Mounting evidence supports the critical role of EMT in orchestrating embryogenesis, tissue regeneration, and cancer metastasis [1]–[3], [16], [27], [30]–[35]. Genetic alteration and morphological changes are modulated during cancer metastasis, such as loss of epithelial cell identity (e.g., occludin and EpCAM) and acquisition of mesenchymal features (e.g., vimentin and TWIST1) [1]–[3], [27]. In development, the EMT and the reverse process, MET are an essential mechanism in the regulation of phenotypic plasticity which is characterized as the cellular and molecular events involved in the interconversion between epithelium and mesenchyme during embryonic assembly and remodeling. However, in cancer, phenotypic plasticity could affect tumor progression and drug efficacy by maintaining the mesenchymal, invasive phenotype of tumor cells that have undergone EMT in response to a diverse array of extracellular stimuli or growth derivatives.

Primary epithelial ovarian cancers are for the most part exquisitely sensitive to platinum-based therapies. Furthermore, recurrent disease frequently responds to additional rounds of chemotherapy; however, the progression-free interval becomes shorter after each cycle, as chemoresistance increases until the disease becomes incurable. The mechanisms leading to drug resistance are complex; however, recent studies suggest that EMT is closely associated with response to therapy and overall or progression free survival. It is thought that intrinsic factors derived from the tumor microenvironment induce epithelial OCCs to undergo profound phenotypic conversion from epithelial (round-shaped) to mesenchymal (spindle-shaped) morphology consistent with EMT [16], [27], [30]–[35]. Recent studies have revealed that the OCCs with epithelial features are more sensitive to chemotherapy regimens as compared to mesenchymal tumor cells [1], [2]. However, the epithelial OCCs undergoing EMT are likely to induce acquired resistance and become unresponsive to conventional drug treatments [1]–[3], [35]. Thus, we hypothesize that reversal of EMT phenotype could help overcome platinum-associated resistance.

Few strategies have been utilized to promote the conversion of mesenchymal cancer cells towards a more epithelial-like phenotype as characterized by the loss of mesenchymal phenotypic traits and the acquisition of epithelial markers [28]–[30]. Park and colleagues [28] have shown that ectopic expression microRNAs (miRNAs), such as miR-200, can lead to up-regulation of E-cadherin in cancer cell lines and reduced motility via targeting the E-cadherin transcriptional repressors, *ZEB1* (TCF8/δEF1) and *ZEB2* (SMAD-interacting protein 1 [SIP1]/ZFXH1B) transcripts. They also reported a significant correlation between E-cadherin and miR-200 expression in two sets of patient samples derived from primary serous papillary ovarian cancer [28]. Similar results have been reported when miR-429 is overexpressed in metastatic ovarian cancer cells [29]. Others have generated a gain-of-function phenotype using transfection with E-cadherin in mesenchymal IOSE-29 cells to restore epithelial properties of normal ovarian surface epithelium and restrict their mesenchymal potential [30]. We therefore explored a more direct approach to induce MET, i.e., treatment with an extracellular morphogen, epimorphin, to revert the OCCs with mesenchymal traits to a more epithelial-like state where they can be more effectively treated with existing chemotherapies.

Like other morphogens activin, bone morphogenetic protein (Bmp) 4 and Sonic Hedgehog (Shh), epimorphin appears to function as graded signals that control gene expression changes and cell fate selection in a concentration-dependent manner. Here, we report that exogenous epimorphin was able to substantially shift ovarian cancer cells with mesenchymal phenotypes to those with epithelial-like features. Our data also provide evidence that pharmacologic doses of epimorphin are critical in determining mesenchymal versus epithelial cell lineage selection in the OCCs. In our studies, exogenous epimorphin showed a dose-dependent activity, in which higher doses (20 µg/ml) of the ligand could promote the conversion to a more epithelial-like phenotype while a two-fold decrease in concentration (10 µg/ml) helped retain a mesenchymal lineage. Experimental evidence from tissues in both vertebrates and invertebrates indicates that morphogens could direct the differentiation of two distinct cell types with relatively small, two- to three-fold, changes in concentration being sufficient to switch cells between alternative fates [37]. To understand a two-fold difference in epimorphin concentration could alter epigenetic controls in the OCCs is intriguing. Evidence suggests that a mechanism for producing discrete cell fate decisions is due to differences in ligand concentration perceived intracellularly as qualitative differences in signal transduction via distinct types of receptors to initiate different downstream signaling events [37]. Therefore, we believe that the absolute number of αV-integrin receptors activated by epimorphin determines the cell lineage selection and perhaps a two-fold difference in the number of αV-integrin receptors is sufficient to specify distinct epithelial gene expression phenotypes in the OCCs.

We also showed that the acquisition of epithelial phenotypes is linked with activation of C/EBPβ and KLF4, downstream transcription factors of αV integrin receptors [12], [15], [17]. Based on our data that epimorphin-specific mediators, C/EBPβ and, to a greater degree, KLF4, increase in response to epimorphin, we believe that the epithelial induction that occurs with higher doses of epimorphin is due to increased epimorphin pathway activation, that then serves as a competitive inhibitor of mesenchymal signaling. Along these lines, others have reported that epimorphin could mediate epithelial morphogenesis by the activation of αV integrin receptors and C/EBPβ in mammary and intestinal cells [10], [12], [31].

The observed pro-epithelial effect upon treatment of OCCs with epimorphin is of interest, not only with respect to their drug sensitivity, but also because of implications regarding mechanisms contributing to cellular differentiation. Thus, further analysis of expression patterns of epimorphin regulatory genes will be critical to delineate more closely the cell lineage commitment. We found that expression of αV-integrin receptors and their downstream effector, C/EBPβ [12], was significantly enhanced in A1847 following treatment with higher-dose epimorphin, whereas TWIST1 was suppressed. Surprisingly, KLF4 mRNAs, a transcriptional regulator of proliferation and differentiation in epithelial cells, both during development and tumorigenesis [16]–[18], were elevated 25- to 30-fold. These results are consistent with a paradigm in which C/EBPβ and KLF4 are important for epithelial-like differentiation [16]–[19], [32]. The commitment to epithelial cell lineage upon stimulation of ovarian tumor cells with an optimal dose of epimorphin to induce MET leads to enhanced expression of several epithelial markers (e.g., β-catenin, occludin and EpCAM) and downregulation of mesenchymal markers (e.g., vimentin, dystroglycan, and palladin). Similar pro-epithelial effects of epimorphin were confirmed using immunostaining for β-catenin and immunoblotting for occludin, CK-19 and mucin-1. Again, we observed a “dosage window” phenomenon in which 10 µg/ml promoted a mesenchymal-like morphology and led to induction of MMP3 while 20 µg/ml favor epithelial-like features and led to concomitant elevation of laminin secretion. Together, these observations suggest that the OCCs require an optimal concentration of epimorphin in order to generate distinct transcriptional responses. Furthermore, we demonstrated that when the OCCs undergo epimorphin-induced MET, they become more sensitive to the cytotoxic effects of platinum. The result presented in this study for the first time suggested that treatment of the OCCs with optimal doses of epimorphin to induced MET could result in re-sensitization of tumor cells to platinum-based therapies.

In summary, these studies demonstrate that exposure to exogenous epimorphin is capable of inducing a shift towards a more epithelial-like phenotype in the OCCs exhibiting mesenchymal features. This epimorphin-induced epithelial phenotypic commitment resulted in enhanced sensitivity to carboplatin, an effective frontline therapy that ultimately fails in the recurrent disease setting. The current data have important implications regarding overcoming platinum resistance, which contributes to the overall poor survival of women diagnosed with advance staged disease. These studies also lay the foundation for future studies to better delineate the potential mechanisms by which epimorphin induces MET and partially restores drug sensitivity. Overall, these studies propose that epimorphin activates αV integrin receptors and the transcription factors C/EBPβ and KLF4 which can be a positive regulator of endogenous epithelial differentiation in ovarian cancer cells.

## Supporting Information

Figure S1 **Analysis of expression of EMT-associated proteins.** Protein expression of the mesenchymal-associated markers, e.g., vimentin and palladin were evaluated by immunoblotting and band densities were quantified by Image-J software. Fold increases in protein expression of treated (10 µg/ml epimorphin) versus untreated A1847: 2-fold increase for vimentin; and 1-fold increase for palladin. β-actin served as the loading control. (means ± S.D., n = 3). [*p<0.05 compared with control].
(TIF)

Figure S2 **EpCAM activation of A1847 in response to epimorphin.** FACS analysis was used to assess the number of EpCAM-positive cells following exposure to epimorphin. A-C: To evaluate EpCAM expression, epimorphin-treated and untreated A1847 cells were permeabilized and stained with anti-EpCAM-FITC conjugate. As shown, treatment with epimorphin (20 µg/ml) (B) leads to a 3-fold increase in EpCAM positive cells as compared to untreated A1847 (A). Histogram overlay analysis was performed using FlowJo software (C). Profiles presented in panels A–B are representative of 3 independent experiments.
(TIF)

Figure S3 **Vimentin suppression of A1847 in response to epimorphin.** FACS analysis was used to assess the number of vimentin-positive cells following exposure to epimorphin. To evaluate vimentin expression, epimorphin-treated and untreated A1847 cells were permeabilized and stained with anti-vimentin-FITC conjugate. As shown, treatment with epimorphin (20 µg/ml) (B) leads to a 1-fold decrease in vimentin positive cells as compared to untreated A1847 (A). Histogram overlay analysis was performed using FlowJo software (C). Profiles presented in panels A–B are representative of 3 independent experiments.
(TIF)

Figure S4 **Carboplatin-induced changes in cell viability following epimorphin-induced EMT.** A–C: A1847, A2780, and OVCAR10 were treated with 10 µg/mL epimorphin for 3 days. After 3 days, epimorphin-treated and untreated OCCs were cultured in triplicate with serial doses of carboplatin for an additional 3 days. Cell viability was quantified using a CellTiter Blue® assay (A–C). A–C: IC_50_ values to carboplatin indicate more cell viability gain in all three epimophin-treated OCCs than the untreated controls in a dose-dependent manner.
(TIF)

Table S1 **PCR Primer Sequences.**
(DOCX)
