# Correction: Epimorphin-Induced MET Sensitizes Ovarian Cancer Cells to Platinum

**DOI:** 10.1371/annotation/9c557fb1-7997-4c21-bd11-03bf5c3d4418

**Published:** 2013-10-01

**Authors:** Kok-Hooi Yew, Jennifer Crow, Jeff Hirst, Ziyan Pessetto, Andrew K. Godwin

The name of the fourth author is incorrect. The correct name is: Ziyan Pessetto. 

The correct citation is: Yew K-H, Crow J, Hirst J, Pessetto Z, Godwin AK (2013) Epimorphin-Induced MET Sensitizes Ovarian Cancer Cells to Platinum. PLoS ONE 8(9): e72637. doi:10.1371/journal.pone.0072637. 

