# Supplementary figures and images for: Epimorphin-Induced MET Sensitizes Ovarian Cancer Cells to Platinum
Source: PLoS One. 2013 Sep 9;8(9):e72637. doi: 10.1371/journal.pone.0072637 (PMC3767807; doi:10.1371/journal.pone.0072637)

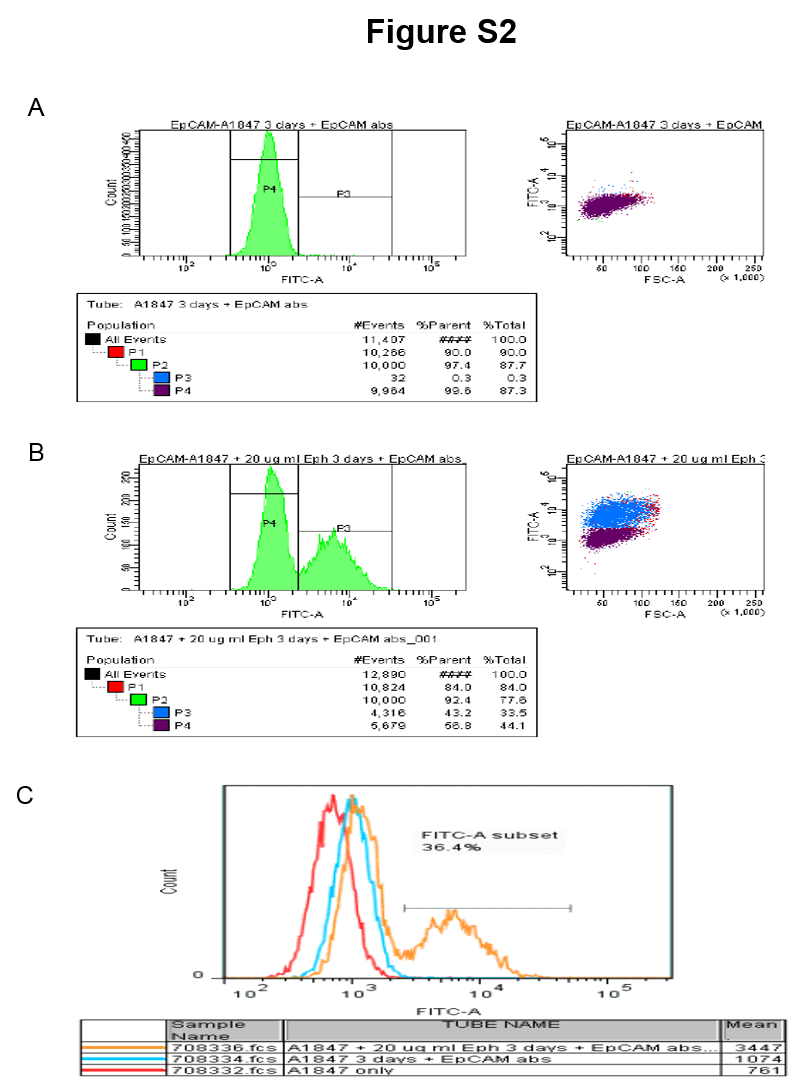

Supplement: Figure S2 — EpCAM activation of A1847 in response to epimorphin. FACS analysis was used to assess the number of EpCAM-positive cells following exposure to epimorphin. A-C: To evaluate EpCAM expression, epimorphin-treated and untreated A1847 cells were permeabilized and stained with anti-EpCAM-FITC conjugate. As shown, treatment with epimorphin (20 µg/ml) (B) leads to a 3-fold increase in EpCAM positive cells as compared to untreated A1847 (A). Histogram overlay analysis was performed using FlowJo software (C). Profiles presented in panels A–B are representative of 3 independent experiments. (TIF) [file pone.0072637.s002.tif]

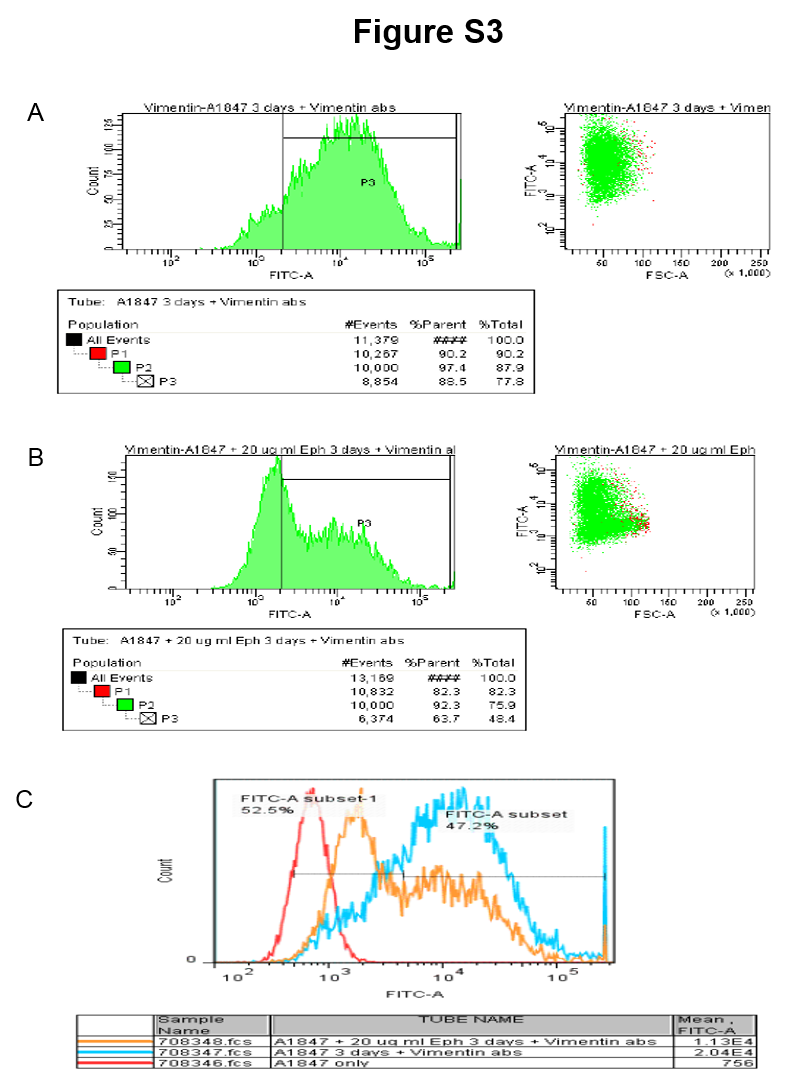

Supplement: Figure S3 — Vimentin suppression of A1847 in response to epimorphin. FACS analysis was used to assess the number of vimentin-positive cells following exposure to epimorphin. To evaluate vimentin expression, epimorphin-treated and untreated A1847 cells were permeabilized and stained with anti-vimentin-FITC conjugate. As shown, treatment with epimorphin (20 µg/ml) (B) leads to a 1-fold decrease in vimentin positive cells as compared to untreated A1847 (A). Histogram overlay analysis was performed using FlowJo software (C). Profiles presented in panels A–B are representative of 3 independent experiments. (TIF) [file pone.0072637.s003.tif]

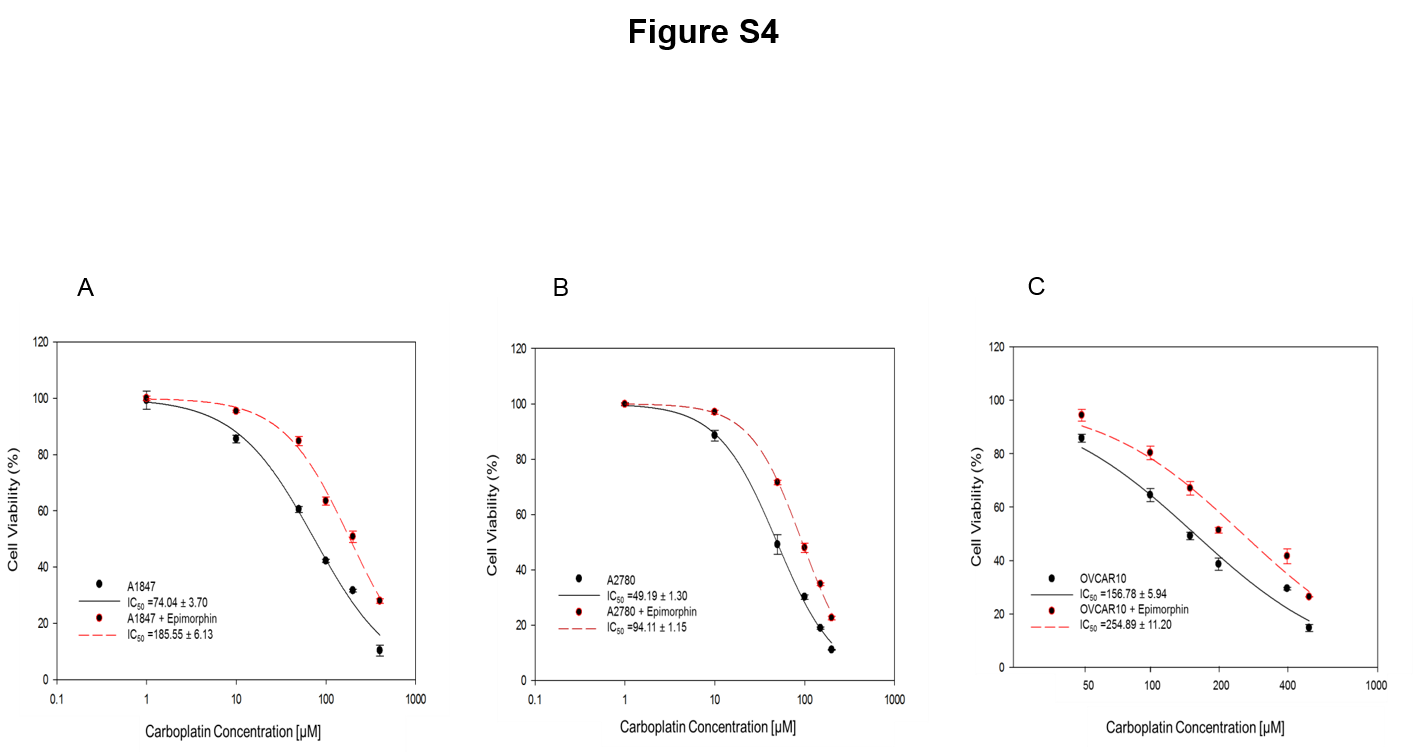

Supplement: Figure S4 — Carboplatin-induced changes in cell viability following epimorphin-induced EMT. A–C: A1847, A2780, and OVCAR10 were treated with 10 µg/mL epimorphin for 3 days. After 3 days, epimorphin-treated and untreated OCCs were cultured in triplicate with serial doses of carboplatin for an additional 3 days. Cell viability was quantified using a CellTiter Blue® assay (A–C). A–C: IC50 values to carboplatin indicate more cell viability gain in all three epimophin-treated OCCs than the untreated controls in a dose-dependent manner. (TIF) [file pone.0072637.s004.tif]
