# Supplementary material for: Epimorphin-Induced MET Sensitizes Ovarian Cancer Cells to Platinum
Source: PLoS One. 2013 Sep 9;8(9):e72637. doi: 10.1371/journal.pone.0072637 (PMC3767807; doi:10.1371/journal.pone.0072637)
Supplement: Table S1 — PCR Primer Sequences. (DOCX) [file pone.0072637.s005.docx]

**Table S1: PCR Primer Sequences**

**Genes** **GenBank Accession #** **Primer Sets** **Product Size (bp)**

PRL13A NM_012423 FP: TGGACCGTCTCAAGGTGTTT

RP: CCCCAGATAGGCAAACTTTC 122

αV integrin NM_002210 FP: TTCTTCCGATTCCAAACTGG

RP: TGCCTTGCTGAATGAACTTG 119

C/EBPβ NM_005194 FP: GACAAGCACAGCGACGAGTA

RP: AGCTGCTCCACCTTCTTCTG 158

KLF4 NM_004235 FP: CCCACACAGGTGAGAAACCT

RP: ATGTGTAAGGCGAGGTGGTC 169

β-catenin NM_001098209 FP: GAAACGGCTTTCAGTTGAGC

RP: CTGGCCATATCCACCAGAGT 166

Occludin NM_001205254 FP: TCCAATGGCAAAGTGAATGA

RP: GCAGGTGCTCTTTTTGAAGG 170

EpCAM NM_002354 FP: TGCAGGGTCTAAAAGCTGGT

RP: ATGCATCTCACCCATCTCCT 146

TWIST1 NM_000474 FP: GTCCGCAGTCTTACGAGGAG

RP: CCAGCTTGAGGGTCTGAATC 159

Vimentin NM_003380 FP: GAGAACTTTGCCGTTGAAGC

RP: CTCAATGTCAAGGGCCATCT 144

Dystroglycan NM_001165928 FP: TCCAGTCAGGGATCCTGTTC

RP: CAACTGCAGTAGGCTCCACA 182

Palladin NM_001166108 FP: TCCGATGAGGAAATTCAAGG

RP: TCGACGAGCCATTCTTTCTT 129

(FP = Forward Primer; RP = Reverse Primer; bp = basepair)
